# Diagnostic value of inflammatory biomarkers of IgA vasculitis based on Olink technology

**DOI:** 10.3389/fimmu.2026.1810681

**Published:** 2026-05-14

**Authors:** Jin-Yue Huang, Zi-Bo Zhang, Ning Wang, Hui Ma, Li Liu

**Affiliations:** 1 Children’s Hospital, Tianjin University (Tianjin Children’s Hospital), Tianjin, China; 2 Tianjin Institute of Pediatrics, Tianjin, China; 3 Tianjin Key Laboratory of Birth Defects for Prevention and Treatment of Child Birth Defects, Tianjin, China; 4 Department of General Internal Medicine, Immunology Direction, Children’s Hospital, Tianjin University (Tianjin Children’s Hospital), Tianjin, China; 5 Department of Laboratory Department Children’s Hospital, Tianjin University (Tianjin Children’s Hospital), Tianjin, China

**Keywords:** biomarkers, diagnosis, IgA vasculitis, inflammation, Olink

## Abstract

**Background:**

Abdominal IgA vasculitis (IgAV) often presents with nonspecific symptoms, complicating accurate diagnosis. There is an urgent need for plasma biomarkers that can reliably distinguish affected patients from healthy individuals.

**Objective:**

To identify and validate plasma inflammatory biomarkers for abdominal IgAV using Olink technology.

**Methods:**

Fasting plasma samples were collected from 10 IgAV patients with abdominal involvement and 10 healthy controls. The Olink Target 96 Inflammation panel was employed to quantify 92 inflammation-related proteins, followed by enzyme-linked immunosorbent assay (ELISA) validation in an expanded cohort.

**Results:**

Twenty-two cytokines were significantly upregulated in IgAV patients compared to controls. Among these, IL-8, OSM, TGF-α, TNFSF-14, and HGF exhibited strong diagnostic potential. In the validation cohort, IL-8, OSM, and TNFSF-14 remained significantly elevated. The combined ROC AUC for these three markers was 0.922 (95% CI: 0.823–1.021), with a sensitivity of 83.3% and specificity of 90.0%, notably outperforming any single marker. Decision curve analysis confirmed the clinical utility of this combined panel. Functional enrichment analysis revealed that these proteins are closely linked to inflammatory pathways, including cytokine-cytokine receptor interaction and the NF-κB signaling cascade.

**Conclusion:**

IL-8, OSM, and TNFSF-14 represent promising plasma biomarkers for abdominal IgAV. Our findings offer novel insights into disease pathogenesis and indicate a more accessible diagnostic strategy.

## 1 Introduction

IgA vasculitis (IgAV), formerly known as Henoch-Schönlein purpura, is a systemic small-vessel vasculitis characterized by IgA immune complex deposition. It is the most common vasculitis in children, with an estimated annual incidence ranging from 1 in 6,660 to 1 in 4,880, 150 to 205 times higher than that observed in adults (1). The peak incidence occurs during autumn and winter, suggesting possible climate-related environmental triggers. Classic manifestations of IgAV include palpable purpura, arthritis or arthralgia, abdominal pain, and hematuria or proteinuria. Although the disease is often self-limiting, recurrence is common. Gastrointestinal involvement occurs in 10–40% of patients, while renal involvement ranges from 10–55%; these factors constitute the primary contributors to morbidity and mortality (2). Notably, digestive tract involvement is closely associated with renal involvement, and severe gastrointestinal complications such as bleeding, can exacerbate short-term outcomes, whereas renal damage affects long-term prognosis (3).

In clinical practice, early and differential diagnosis of IgAV remains challenging. Conventional diagnostic methods rely on tissue biopsy and classic symptomatology; however, the clinical picture is often heterogeneous and non-specific. This is pronounced for the abdominal phenotype: when abdominal pain is the presenting symptom, it can be easily misdiagnosed as surgical acute abdomen, leading to delayed or missed diagnosis (4). Currently, no convenient, sensitive, or broadly applicable plasma biomarkers exist for early IgAV screening. Identifying reliable biomarkers that can distinctly separate abdominal IgAV patients from healthy individuals would, therefore, hold significant clinical value for timely recognition and treatment optimization.

Prior research indicates that IgAV arises when environmental triggers such as infections interact with genetically susceptible individuals, leading to dysregulated immune responses and systemic small-vessel inflammation (5). Inflammatory cytokines are hypothesized to play a central role in this process (6). Multiple studies have documented elevated levels of various cytokines (e.g., IL-6, TGF-α) in both peripheral blood and affected tissues of IgAV patients, with certain cytokine levels correlating with disease activity and organ involvement (7, 8). These findings support the exploration of inflammation-related proteins as potential biomarkers. However, systematic high-throughput proteomic screening specific to abdominal IgAV remains absent, and the diagnostic performance and clinical applicability of candidate biomarkers have yet to be established.

Recent advancements in proteomics have rendered high-throughput screening more accessible. The proximity extension assay (PEA), as applied on the Olink platform, offers a robust combination of high sensitivity, specificity, and comprehensive protein coverage (9, 10). This approach facilitates the simultaneous quantification of dozens to hundreds of inflammation-related proteins, substantially expanding the scope for biomarker discovery (11, 12). Furthermore, the platform exhibits excellent reproducibility and requires minimal sample volume, making it particularly suitable for smaller disease cohorts (13).

In this study, we collected fasting plasma samples from patients with abdominal IgAV and healthy controls. Using the Olink platform, we performed high-throughput quantitative analysis of 92 inflammation-related proteins with aims to: (i) identify biomarkers capable of discriminating abdominal IgAV patients from healthy individuals and (ii) map these candidates onto inflammatory pathways and cytokine networks. Through systematic proteomic screening and functional enrichment analysis, we hope to establish a foundation for improved early diagnosis, biomarker development, and mechanistic understanding of IgAV, especially in patients with abdominal involvement.

## 2 Materials and methods

### 2.1 Sample collection and study design

**Figure 1:**
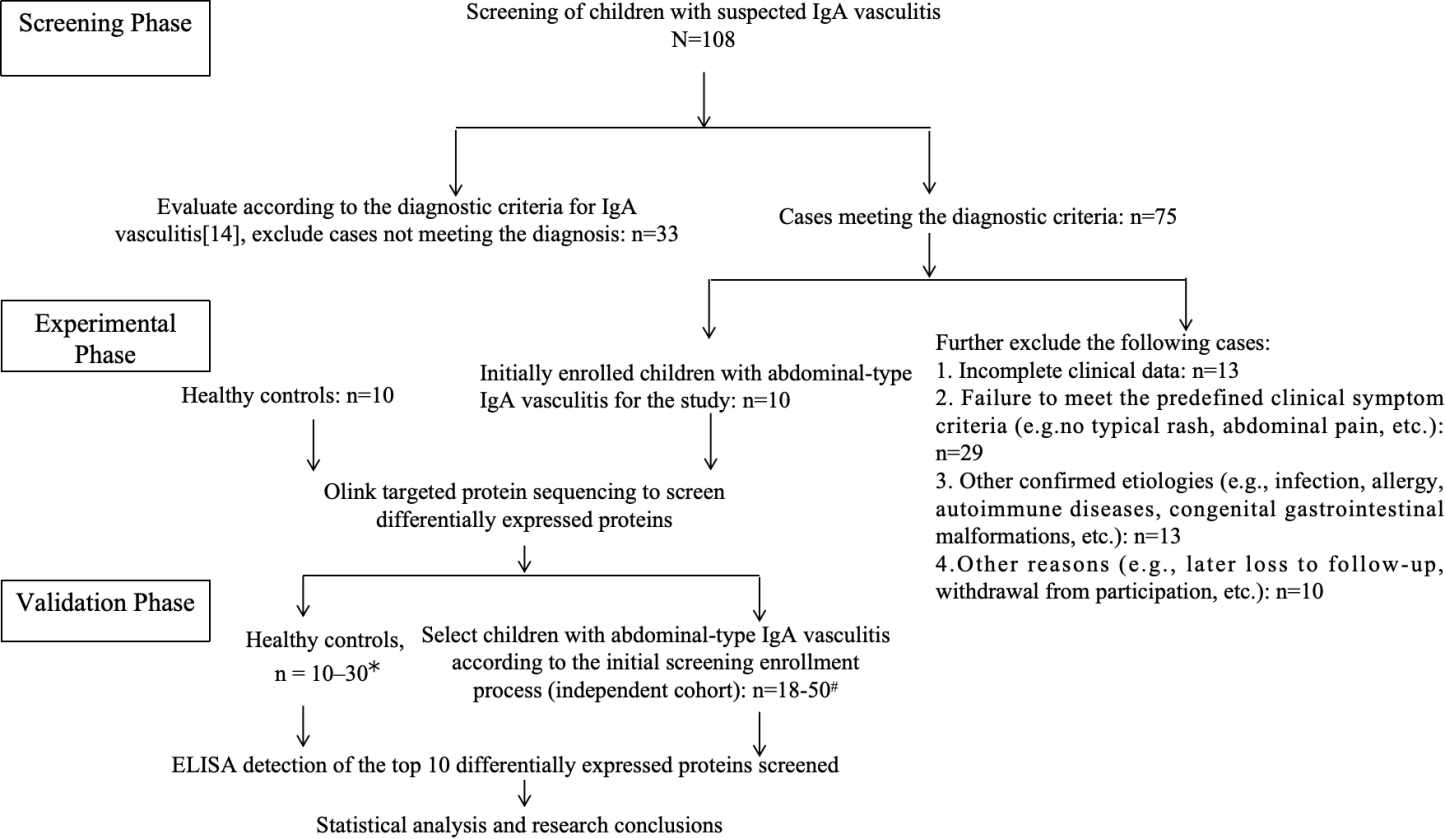
Patient flow diagram according to STARD 2015 guidelines for this diagnostic accuracy study of abdominal-type IgA vasculitis in children. * Three independent external validation cohorts were controlled for external validation. Validation cohort 1: 50 cases and 30 healthy controls; Validation cohort 2: 18 cases and 10 healthy controls; Validation cohort 3: 19 cases and 10 healthy controls.

This study was approved by the Medical Ethics Committee of Tianjin Children’s Hospital (Approval No. 2023-LXKY-008) and conducted in accordance with the Declaration of Helsinki. Written informed consent was obtained from the guardians of all participants. **Figure 1** shows the patient flow diagram created in accordance with STARD 2015 guidelines.

Fasting venous blood samples were obtained on the initial morning of hospitalization. Peripheral blood was collected into EDTA anticoagulant tubes and processed within two hours. Plasma was separated by centrifugation at 1600g for 10 minutes at 4 °C, aliquoted into 2−mL cryotubes, and stored at –80 °C until analysis.

Participants were divided into two groups (n = 10 each): (1) abdominal IgAV and (2) age−matched healthy children recruited from routine physical examinations during the same period (control group). Diagnostic and inclusion criteria followed the 2008 EULAR/PRINTO/PRES guidelines for IgAV (14). Non−thrombocytopenic purpura was mandatory, accompanied by at least one of the following criteria: (1) Acute abdominal pain, including intussusception or gastrointestinal bleeding; (2) Histopathology showing leukocytoclastic vasculitis with IgA deposition or proliferative glomerulonephritis with IgA deposition; (3). Arthritis (acute joint swelling/pain with motion limitation) or arthralgia (acute joint pain without swelling/limitation); (4). Renal involvement: 24−hour urinary protein > 0.3 g, morning urine albumin/creatinine ratio > 30 mmol/mg, or hematuria (>5 Red blood cell/HPF) with red cell casts.

Mixed IgAV was defined as skin purpura plus at least two of the following: arthritis/arthralgia, abdominal pain, or renal involvement. The diagnosis of abdominal IgAV was made based on: (1) Gastrointestinal symptoms (diarrhea, abdominal pain) presented initially; (2) Gastroscopy revealing purpuric lesions, erosions, or ulcers in the gastrointestinal mucosa; and (3) Abdominal CT demonstrating multifocal segmental intestinal involvement, narrowed segments, edematous bowel wall thickening, and often a small amount of ascites.

Exclusion criteria included: (1) Incomplete clinical data; (2) Prior immunosuppressant or glucocorticoid use; (3) Pre-existing digestive disorders (malnutrition, gastrointestinal malformations, peptic ulcers, infections, ulcerative colitis, etc.); and (4) Other rheumatic/autoimmune diseases (Kawasaki disease, rheumatic fever, SLE, IgG4-related disease), endocrinopathies, genetic metabolic disorders, hematologic diseases, or malignancies.

All patients and control subjects underwent evaluations following a standardized clinical protocol specifically aimed at abdominal IgAV. For the case group, diagnostic assessments included evaluation of gastrointestinal symptoms, B-ultrasound, and when clinically indicated, abdominal computed tomography (CT) and/or gastroscopy. All findings were interpreted according to the same predefined diagnostic criteria for abdominal IgAV. For healthy controls, invasive or radiological examinations (such as gastroscopy and abdominal CT) were not performed, as these are unnecessary for these individuals. Instead, standardized history taking and physical examinations were used to confirm the absence of gastrointestinal symptoms, abdominal signs, and relevant diseases. This approach ensures consistent evaluation standards between groups and avoids differential verification bias.

Patients were sequentially enrolled and assigned into discovery and validation cohorts based on a temporal cut-off of December 31, 2024. To ensure independence, no patient enrolled before the cut-off date was included in the validation cohort, and vice versa.

### 2.2 Protein quantification

Protein levels were assessed using the Olink Target 96 Inflammation panel (Olink Proteomics, Uppsala, Sweden) according to the manufacturer’s instructions. This assay, based on PEA technology, allows for the simultaneous quantification of 92 protein analytes from just 1 μL of sample per well.

In brief, paired oligonucleotide-labeled antibodies bind to their corresponding protein targets. When both antibodies are in proximity, their oligonucleotides hybridize, and upon addition of DNA polymerase, proximity-dependent DNA polymerization generates a unique PCR target sequence. These sequences were detected and quantified using a microfluidic real-time PCR system (Biomark HD, Fluidigm). Raw Ct values underwent quality control and normalization using internal and external controls. Final data are reported as Normalized Protein eXpression (NPX) values, arbitrary units on a log2 scale, wherein higher values indicate greater protein abundance.

### 2.3 Bioinformatics analysis

Differentially expressed proteins (DEPs) were identified using the Olink Analyze R package. Proteins with a p-value < 0.05 were considered statistically significant.

Principal component analysis (PCA) was performed using R (https://www.r-project.org/) to visualize sample clustering and reduce dimensionality. PCA transforms thousands of correlated variables (protein expression levels) into a smaller set of linearly uncorrelated principal components, thereby revealing underlying data structures. The PCA data was preprocessed using the z-score method, including background data from the GO and KEGG databases.

Gene Ontology (GO) annotation was used to classify DEPs by biological process, cellular component, and molecular function. For each GO category, a two-tailed Fisher’s exact test assessed enrichment relative to all identified proteins. Pathway enrichment analysis was executed using the Kyoto Encyclopedia of Genes and Genomes (KEGG) database, again employing a two-tailed Fisher’s exact test, with pathways hierarchically categorized according to KEGG nomenclature.

Protein-protein interaction (PPI) networks were constructed using the STRING database (version 11.5). Enrichment was evaluated against the 92−protein background of the Olink inflammation panel. Spearman correlation analysis was used to examine pairwise expression relationships. Cytoscape (version 3.9.1) was employed for network visualization.

Receiver operating characteristic (ROC) curves were generated using the ROCR R package.

### 2.4 ELISA validation

To validate Olink findings, we expanded the cohort of IgAV patients and healthy controls. Three independent external validation cohorts were enrolled for external validation: Validation cohort 1: 50 cases and 30 healthy controls; Validation cohort 2: 18 cases and 10 healthy controls; Validation cohort 3: 19 cases and 10 healthy controls.

ELISA was performed using commercial kits for TNFSF14 (ELK4478), IL-8 (ELK1159), Oncostatin M (OSM) (ELK2302), TGF-α (ELK1184), and HGF (ELK10180; all from Kehuo Biotechnology, Wuhan, China). All procedures followed the manufacturers’ protocols.

### 2.5 Statistical analysis

Statistical analyses were performed using SPSS 25.0. Normally distributed continuous data are expressed as mean ± standard deviation (SD) and compared using independent-samples t-tests. Categorical data are presented as counts (percentages) and compared utilizing χ² tests. Non-normally distributed continuous data are expressed as median (Q1, Q3) and compared using Mann–Whitney U tests.

ROC curves were constructed using Medcalc to determine optimal cut−off values for predicting abdominal IgAV. A nomogram was built using the rms package in R, and its predictive performance was evaluated by decision curve analysis (DCA).

## 3 Results

### 3.1 Comparison of clinical baseline data

As depicted in **Table 1**, there were no significant differences in the distribution of age and gender between the case groups and control groups of the discovery cohort versus the three validation cohorts (P > 0.05). All case groups presented with typical clinical manifestations of IgAV, and the proportions of gastrointestinal, joint, and renal involvement were consistent across groups (P > 0.05). Clinical, endoscopic, and CT characteristics of patients with abdominal IgAV in the discovery and validation cohorts (**Table 2**). These results indicated that the baseline characteristics of the subjects in the four cohorts were homogeneous and comparable.

**Table 1:** Comparison of baseline characteristics among study cohorts [n(%)].

Characteristic	Discovery cohort	Validation cohort 1	Validation cohort 2	Validation cohort 3	Statistic	P value
Case Group						
n	10	50	18	19	–	–
Age (years)	6.5±1.2	7.1±1.5	6.8±1.3	7.0±1.4	*F*=0.195	0.901
Gender (Male/Female)	6/4	28/22	10/8	11/8	χ²=0.338	0.952
Typical Purpuric Rash	10 (100.0)	50 (100.0)	18 (100.0)	19 (100.0)	–	1.000
Gastrointestinal Involvement (Abdominal Pain/Vomiting)	8 (80.0)	42 (84.0)	15 (83.3)	16 (84.2)	χ²=0.267	0.966
Joint Involvement (Arthralgia/Swelling)	4 (40.0)	18 (36.0)	7 (38.9)	8 (42.1)	χ²=0.471	0.925
Renal Involvement (Hematuria/Proteinuria)	1 (10.0)	6 (12.0)	2 (11.1)	2 (10.5)	χ²=0.186	0.980
Control Group						
n	10	30	10	10	–	–
Age (years)	6.2±1.1	6.8±1.4	6.5±1.2	6.7±1.3	*F*=0.195	0.901
Gender (Male/Female)	5/5	16/14	6/4	5/5	χ²=0.413	0.937

**Table 2:** Clinical, endoscopic, and computed tomography characteristics of patients with abdominal IgAV in the discovery and validation cohorts.

Cohort name	Group	n	Abdominal ultrasound [n(%)]	Gastroscopy/colonoscopy [n(%)]	Abdominal CT [n(%)]	Key imaging/endoscopic findings
Discovery Cohort	Case	10	10 (100.0)	5 (50.0)	3 (30.0)	Cases: Bowel wall thickening, mesenteric lymphadenopathy, small peritoneal effusion.
	Control	10	10 (100.0)	0 (0.0)	0 (0.0)	Controls: No significant abnormalities.
Validation Cohort 1	Case	50	50 (100.0)	25 (50.0)	15 (30.0)	Cases: Bowel wall thickening, mesenteric lymphadenopathy, small peritoneal effusion.
	Control	30	30 (100.0)	0 (0.0)	0 (0.0)	Controls: No significant abnormalities.
Validation Cohort 2	Case	18	18 (100.0)	9 (50.0)	5 (27.8)	Cases: Bowel wall thickening, mesenteric lymphadenopathy, small peritoneal effusion.
	Control	10	10 (100.0)	0 (0.0)	0 (0.0)	Controls: No significant abnormalities.
Validation Cohort 3	Case	19	19 (100.0)	10 (52.6)	6 (31.6)	Cases: Bowel wall thickening, mesenteric lymphadenopathy, small peritoneal effusion.
	Control	10	10 (100.0)	0 (0.0)	0 (0.0)	Controls: No significant abnormalities.

Characteristics of the Discovery(Cases = 10), Validation 1 (Cases = 50). Validation 2 (Cases = 18), and Validation 3 (Cases = 19) cohorts.

### 3.2 Differential expression of inflammation-related proteins

**Figure 2:**
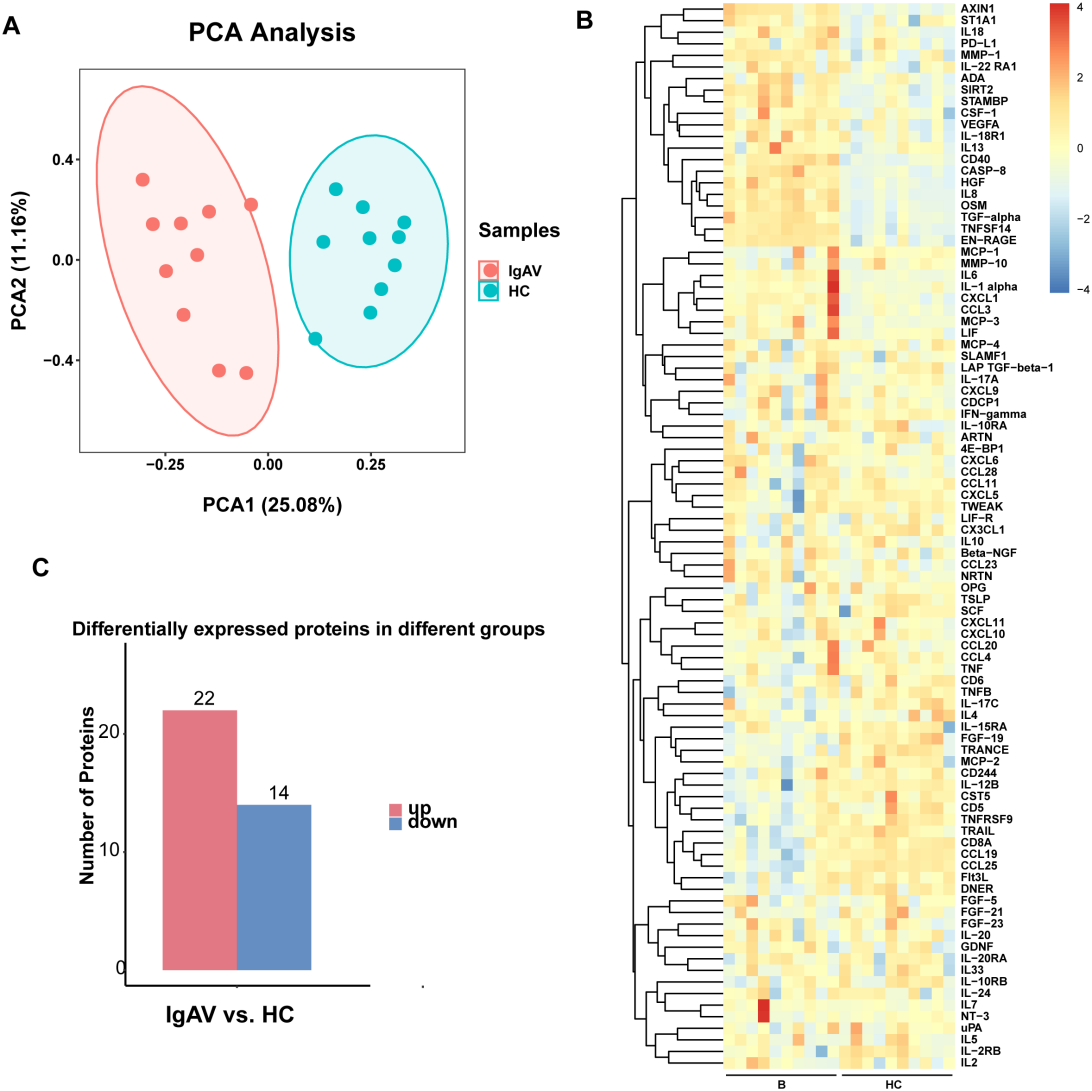
Comparison of serum inflammation- related proteins between the abdominal IgAV group and the healthy control group (Group HC). **(A)** Principal component analysis (PCA) displays two dimensions of the two color-coded populations. Each point represents a patient, with patients exhibiting similar protein expression profiles arranged adjacent to each other. **(B)** Cluster heat map of overall protein expression between the two groups. The x-axis represents the sample group, and the y-axis represents protein names. Different colors indicate varying protein expression levels, with colors ranging from blue to red representing low to high expression levels. **(C)** Comparison of the number of differentially expressed proteins between paired groups.

PCA was used to assess global plasma protein profiles. The two-dimensional scatter plot was constructed using the first principal component (PC1, 25.08%) and the second principal component (PC2, 11.16%). The two groups were clearly separated along the PC1 axis: IgAV samples were mainly distributed in the negative region of PC1, while healthy controls were concentrated in the positive region of PC1, with almost no overlap between the 95% confidence ellipses, indicating significant differences in the overall molecular profiles between the two groups (**Figure 2A**). A heatmap of protein expression is shown in **Figure 2B**. Paired comparison revealed 36 DEPs between the IgAV and control groups, with 14 were downregulated and 22 upregulated (**Figure 2C**).

### 3.3 Key differences between IgAV patients and healthy controls

**Figure 3:**
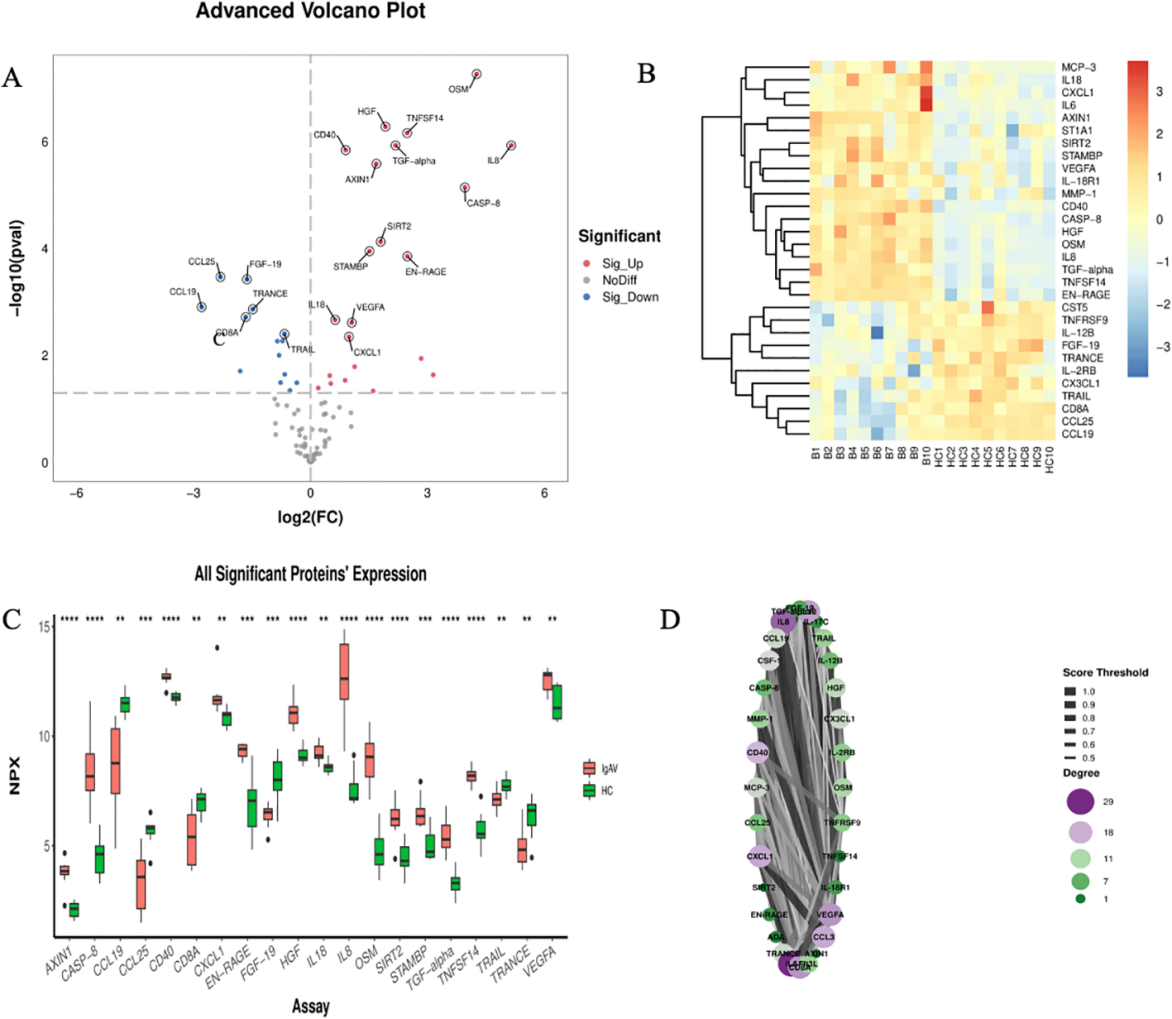
Comparison of serum inflammation-related proteins between the IgAV group and the healthy control group (HC). **(A)** The volcano plot illustrates the differences in protein expression between the IgAV group and the HC group. Proteins with high expression in the IgAV group and HC group are labeled in red and blue, respectively. The x- axis represents the logarithmic fold change (Log2), and the y-axis represents the logarithmic transformation value (-Log10). **(B)** The heat map visually presents the differential expression between the IgAV group and the HC group, with the x-axis representing the sample group and the y-axis representing the protein name. Different colors indicate protein expression levels, ranging from blue (low expression) to red (high expression). The p-value in the volcano plot is the result after undergoing -log10 transformation, and the threshold is set at p < 0.05. **(C)** The box plot displays the differentially expressed proteins between the IgAV group and HC group. **(D)** Protein interaction network. *<0.05, **<0.01, ***<0.001, ****<0.0001.

Olink analysis identified 36 DEPs (p < 0.05). The 20 most significantly altered proteins are annotated in **Figure 3A**, with their expression patterns visualized in the accompanying heatmap (**Figure 3B**) and boxplots (**Figure 3C**). The p-value in the volcano plot reflects results after -log10 transformation, with a threshold set at p < 0.05. PPI network analysis placed IL-6 and IL-8 at the core of the interaction network (**Figure 3D**).

### 3.4 GO and KEGG enrichment analysis

**Figure 4:**
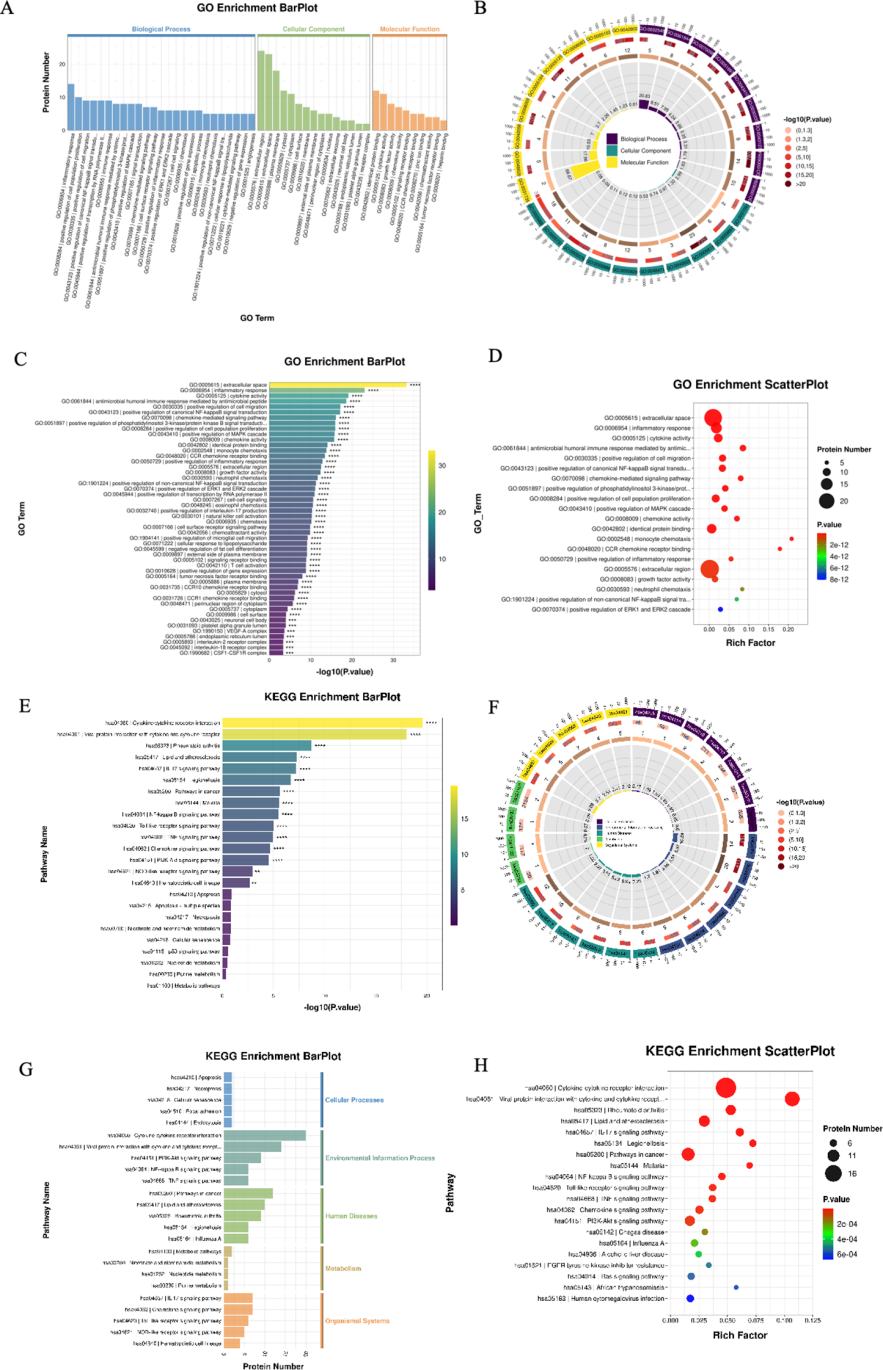
GO **(A-D)** and KEGG pathway analysis **(E-H)** of inflammatory proteins between the IgAV group and the healthy control group (HC).

GO enrichment analysis showed that DEPs were primarily involved in the inflammatory response, positive regulation of cell proliferation, and positive regulation of cell migration (biological processes); localized to the extracellular region, extracellular space, and plasma membrane (cellular components); and associated with protein binding, cytokine activity, and growth factor activity (molecular functions) (**Figures 4A–D**).

KEGG analysis revealed significant enrichment in cytokine-cytokine receptor interaction, viral protein interaction with cytokine and cytokine receptor, and the NF−κB signaling pathway (**Figures 4E–H**).

### 3.5 Candidate biomarker selection

**Figure 5:**
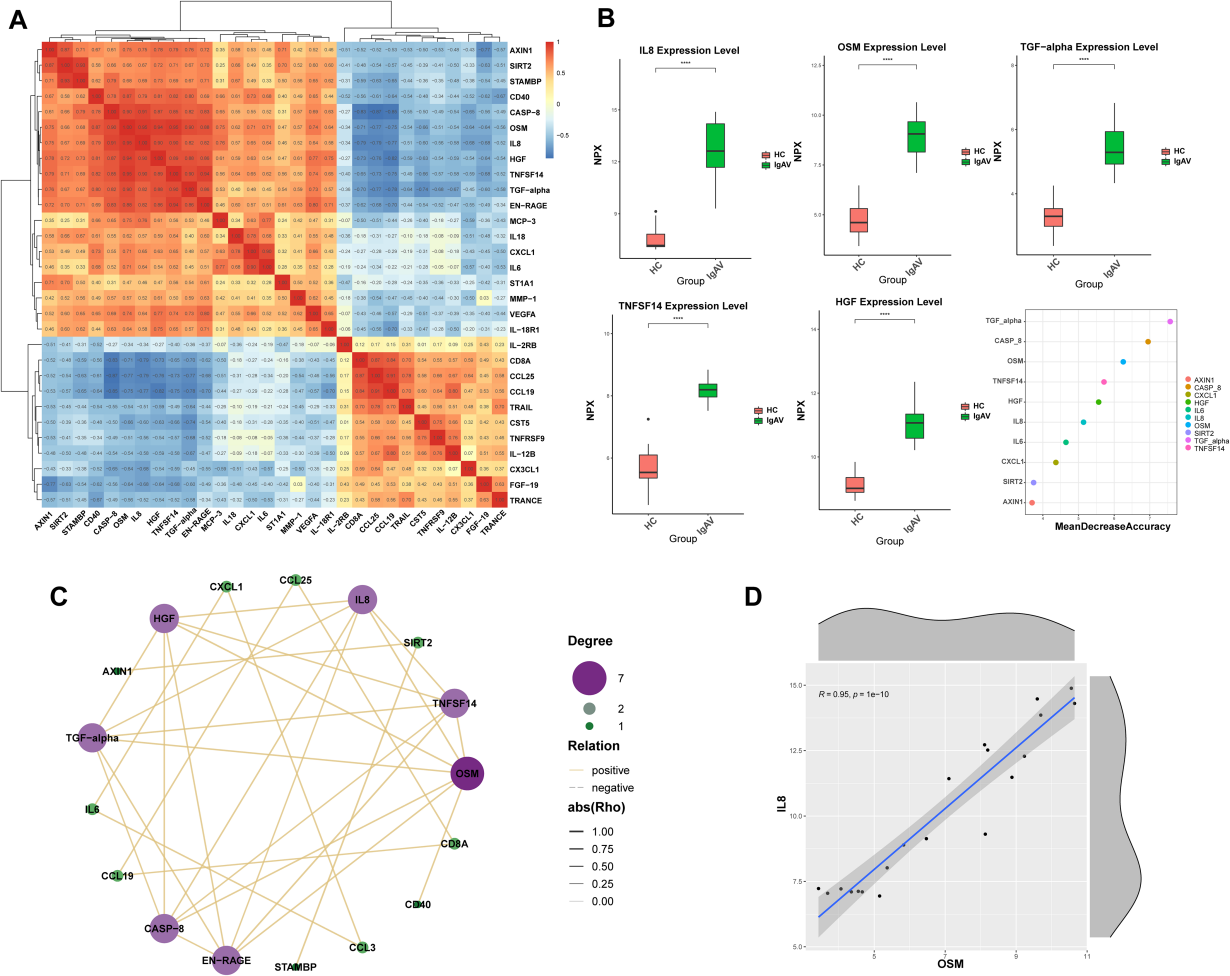
**(A)** Heatmap visually illustrates the differential protein expression between the differential expression group (IgAV group) and the healthy control group (HC group). The x-axis represents sample groups, and the y-axis indicates protein names. Different colors correspond to varying protein expression levels, ranging from blue (low expression) to red (high expression). **(B)** Box plot of significantly differentially expressed proteins between the IgAV group and the healthy control group (HC), The verification was conducted based on the top 10 items in the random forest chart (specifically: IL-8, OSM, TGF-α, TNFSF-14, and HGF) **(C)** Protein interaction network diagram, with IL-8, OSM, TGF-a, TNFSF-14, and HGF as the primary differential proteins, and OSM as the core protein. **(D)** Scatter plot showing the highest significance of IL-8 and OSM.

From the 36 DEPs, we selected 20 with the most pronounced differences, with 14 upregulated and 6 downregulated (**Figure 5A**). ROC analysis was performed for five upregulated candidates: IL-8, OSM, TGF-α, TNFSF-14, and HGF. Random forest analysis demonstrated the top 10 DEPs (**Figure 5B**).

PPI analysis of these five candidates identified OSM as a central node (**Figure 5C**). Correlation analysis showed the strongest pairwise correlation between IL−8 and OSM (**Figure 5E**).

### 3.6 ELISA validation

**Figure 6:**
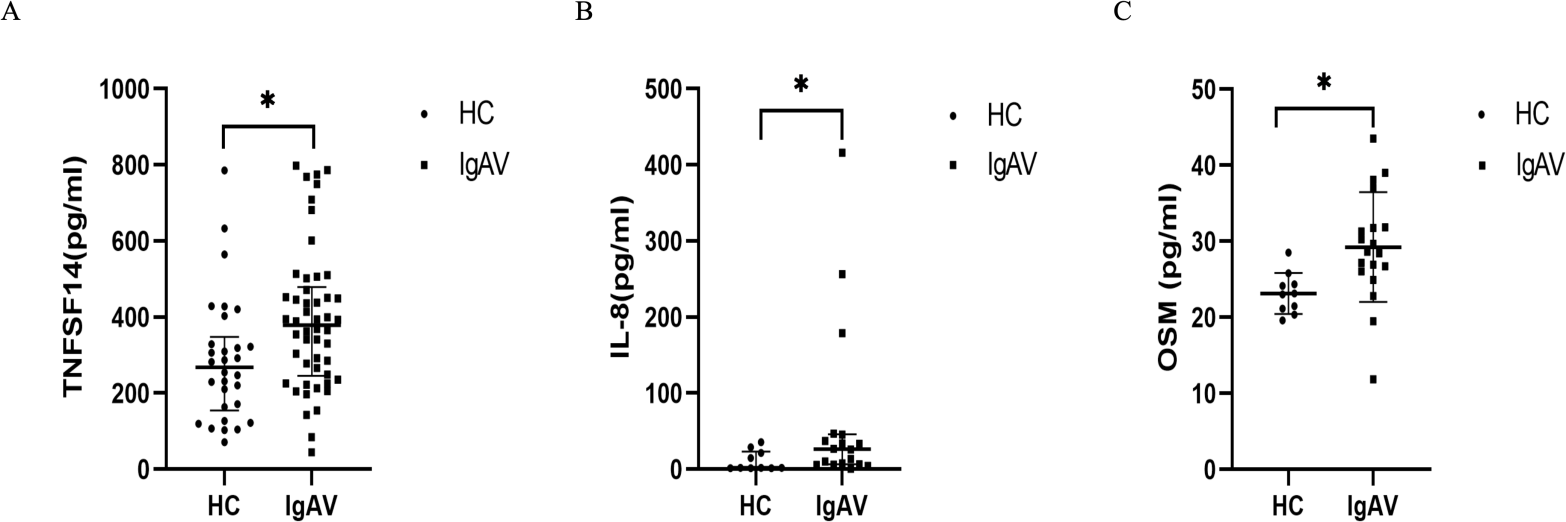
**(A)** TNFSF14; **(B)** IL-8; **(C)** OSM; HC, healthy controls; IgAV, IgAV group **(A)** Data are presented as median with interquartile range (HC:30 cases IgAV: 50 cases **(B)** Data are presented as median with interquartile range (HC:10 cases IgAV: 18cases) **(C)** Data are presented as mean±SD (HC:10 cases IgAV: 19 cases). Symbol * indicates statistically significant differences between groups (p < 0.05).

To validate our Olink findings, we expanded the cohort of IgAV patients and healthy controls. Three independent external validation cohorts were enrolled for external validation: Validation cohort 1: 50 cases and 30 healthy controls; Validation cohort 2: 18 cases and 10 healthy controls; and Validation cohort 3: 19 cases and 10 healthy controls. TGF-α and HGF showed inconsistent results between Olink and ELISA and were therefore not pursued further. In contrast, TNFSF-14, IL-8, and OSM remained significantly elevated in IgAV patients (**Figures 6A–C**), with ROC AUC values consistent with the Olink data. These results confirm that TNFSF-14, IL-8, and OSM are reliably upregulated in abdominal IgAV and may serve as robust diagnostic biomarkers.

### 3.7 Comparisons of predictive performance between single and combined markers

**Figure 7:**
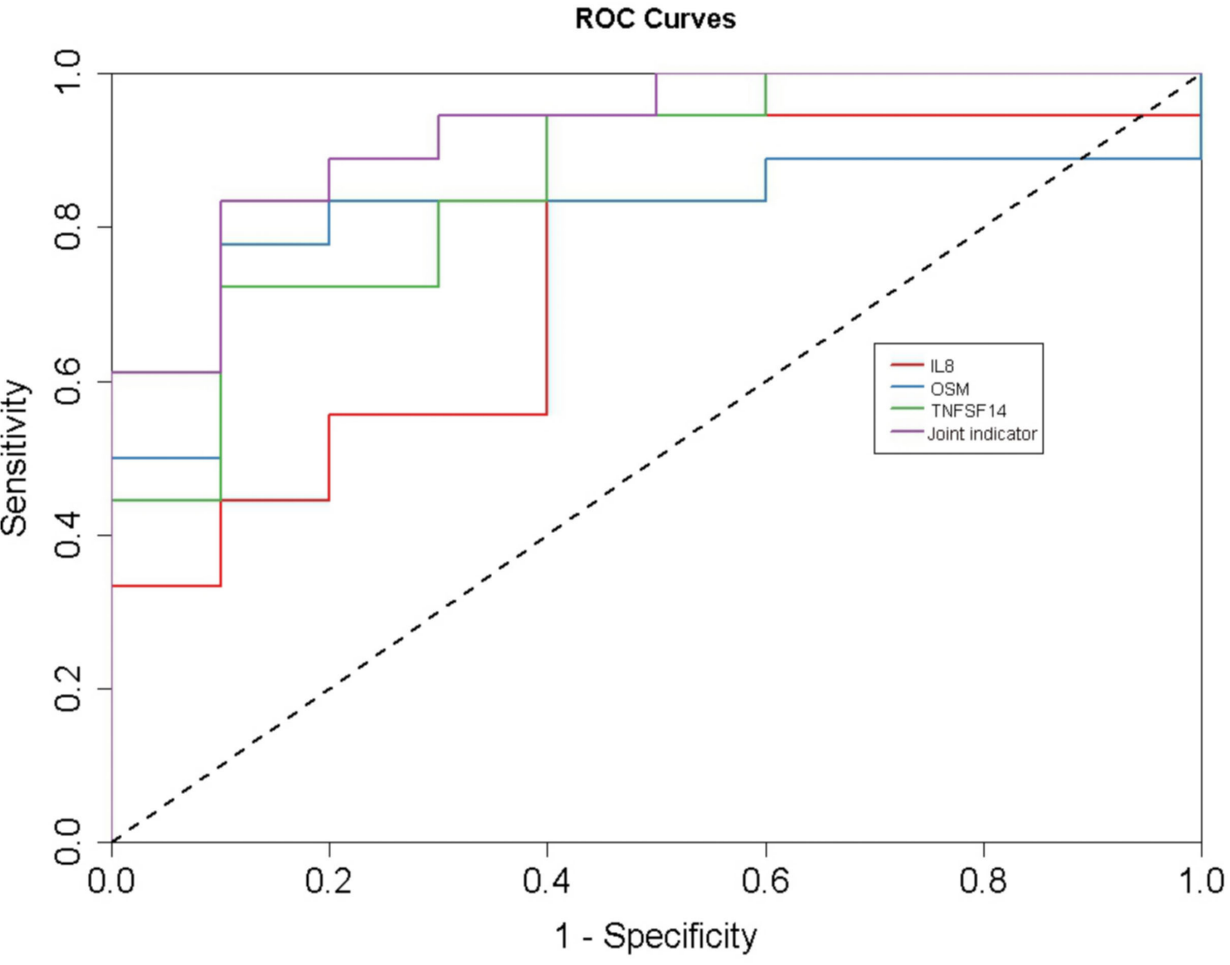
Serum IL-8, OSM, TNFSF14 and combined markers ROC curve for predicting the occurrence of abdominal IgAV(IL8:AUC 0.756,95%CI 0.564-0.947; OSM:AUC 0.817,95%CI 0.651-0.982); TNFSF14:AUC 0.861,95% CI 0.721-1.001; Joint indicator AUC: 0.922, 95% CI 0.823 - 1.021).

ROC curves for IL-8, OSM, TNFSF-14, and their combination are shown in **Figure 6**. The combined panel yielded an AUC of 0.922 (95% CI: 0.823–1.021, p < 0.05). At a cut-off value of 0.650, sensitivity reached 83.3%, specificity 90.0%, and the Youden index was 0.733—all surpassing performance of any single marker (**Table 3**, **Figure 7**).

**Table 3:** ROC curve parameters for serum IL-8, OSM, TNFSF14 and combined markers.

Variables	Cut-off	Sensitivity (%)	Specificity (%)	AUC (95%CI)	P
IL8	3.171	0.944	0.600	0.756(0.564-0.947)	0.027
OSM	25.935	0.778	0.900	0.817(0.651-0.982)	0.006
TNFSF14	329.871	0.722	0.900	0.861(0.721-1.001)	0.002
Joint Indicator Group	0.650	0.833	0.900	0.922(0.823-1.021)	0.000

### 3.8 Nomogram construction

**Figure 8:**
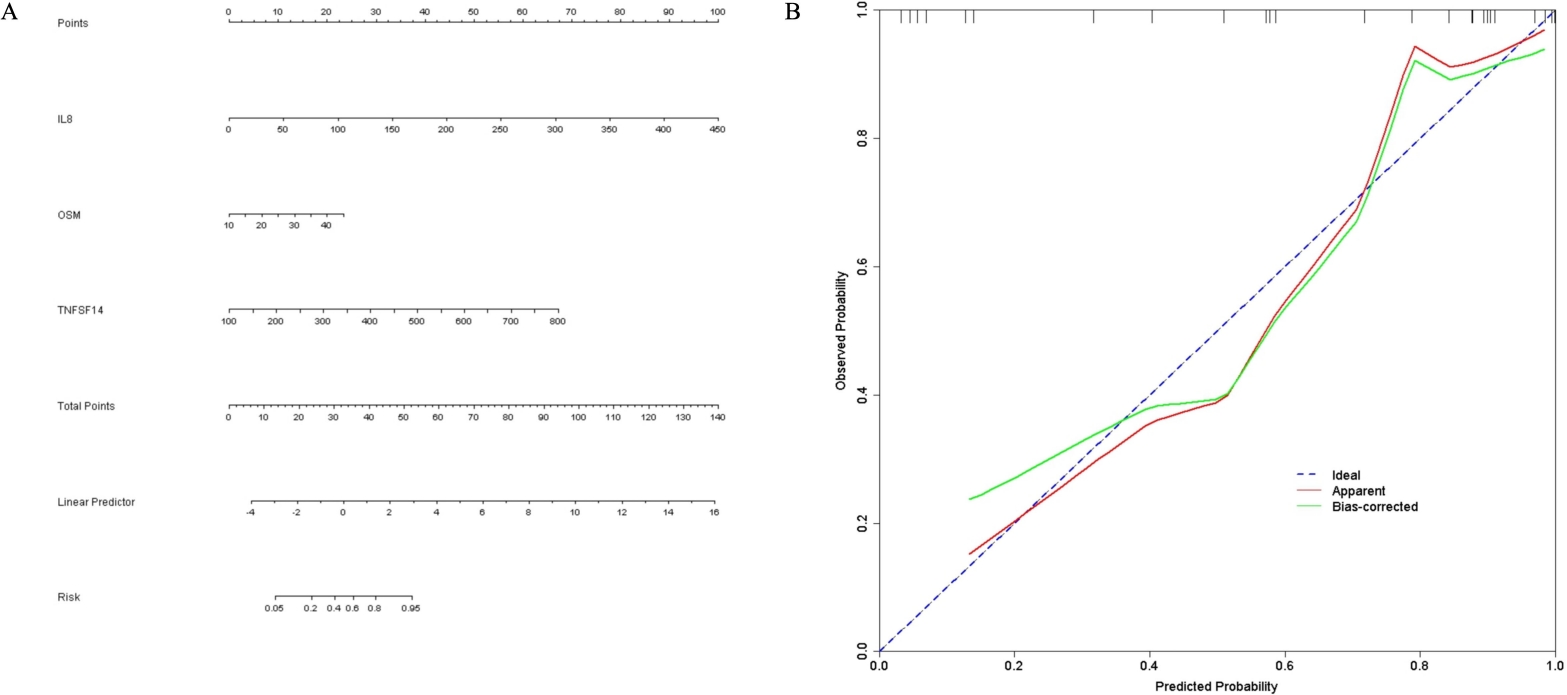
**(A)** Line chart predicting the occurrence of abdominal IgAV based on the combined serum IL-8, OSM, and TNFSF14 marker **(B)** Calibration curve.

A nomogram for predicting abdominal IgAV was developed using logistic regression (**Figure 8A**). Each variable contributed to a score, with the total score corresponding to a predicted risk. For example, a total score of 25–30 indicates a 60.00%–82.00% risk of abdominal IgAV. The calibration curve showed good agreement between predicted and observed probabilities (**Figure 8B**). The blue dotted line represents the ideal calibration state (where predicted probability matches the observed occurrence rate), the red solid line depicts the apparent calibration curve on the modeling dataset, and the green solid line reflects the bias-corrected calibration curve after Bootstrap resampling. In the predictive model for discrimination assessment, the initial C-index was 0.9222, indicating excellent discriminatory ability for the outcome event (evaluation criterion: >0.9 signified excellent discrimination1). To address potential overfitting bias, internal validation was conducted using the Bootstrap method, resulting in a bias-corrected C-index of 0.8873, which still qualifies as adequate (evaluation criterion: >0.8 indicates good performance). This indicates that the model maintained stable discriminatory performance in repeated sampling validation.

### 3.9 Decision curve analysis

**Figure 9:**
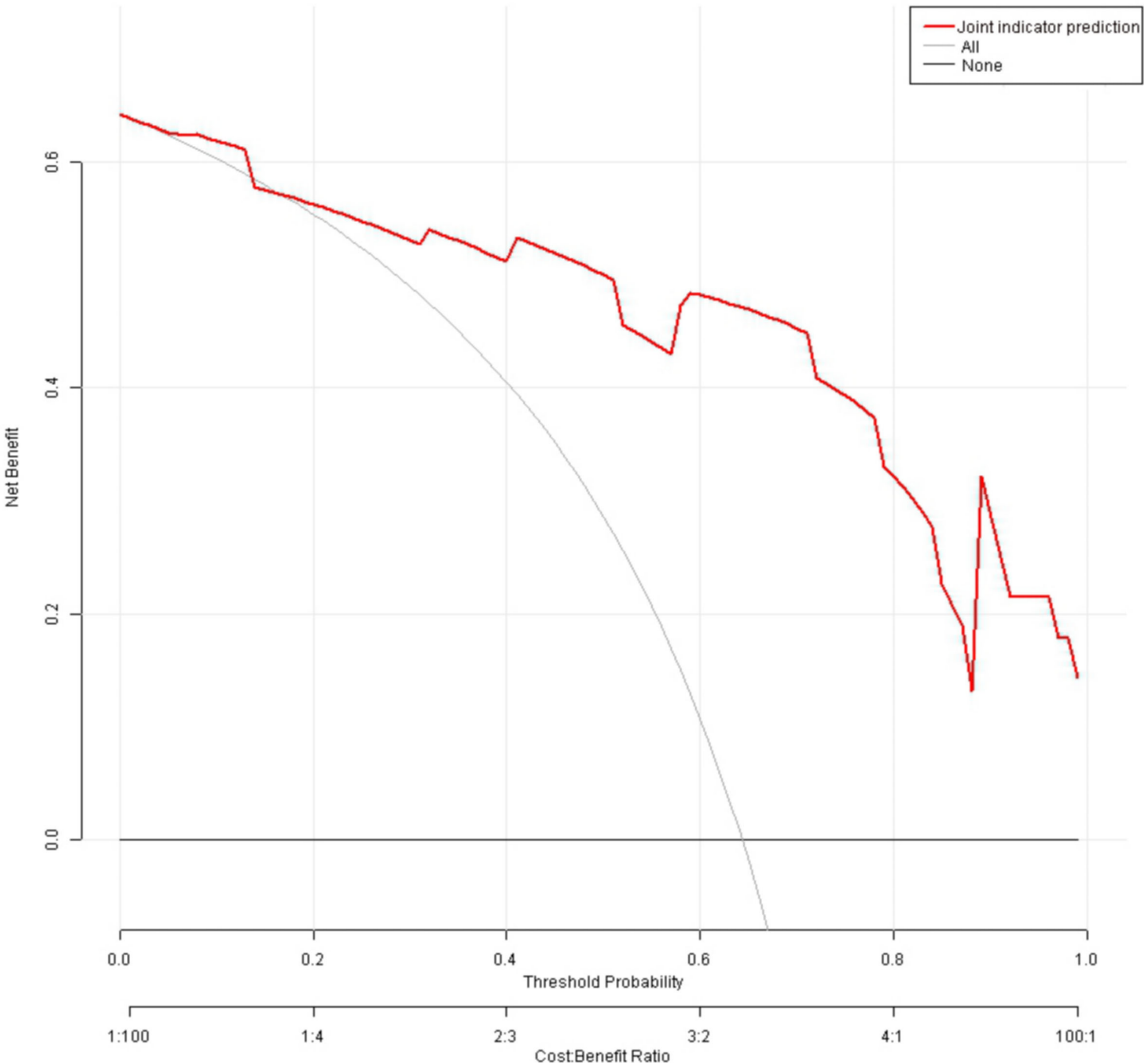
Decision curve for predicting the occurrence of abdominal IgAV using a combination of serum IL-8, OSM, and TNFSF14.

DCA was performed with net benefit on the y-axis and high-risk threshold on the x-axis (range 0–1). Across a broad range of threshold probabilities from approximately 0 to 0.85, the decision curve for the prediction model consistently lied above both the “All” and “None” lines (**Figure 9**). This suggests that within this range, utilizing the nomogram for risk stratification toward guiding decisions yields a higher net benefit compared to either an “intervene on all” or “intervene on none” strategy.

There were fluctuations at high thresholds. In the region where the threshold probability exceeds 0.85, the curve exhibits noticeable fluctuations and a decline. This is likely due to the small sample size of high-risk patients, leading to reduced stability in the estimates. It suggests that at very high-risk thresholds, the clinical applicability of the model is limited.

Regarding clinical applicability, the majority of clinically acceptable risk thresholds (10%–85%), using this nomogram model for risk stratification can prevent 2 to 25 unnecessary interventions or adverse events per 100 treated patients compared to an “intervene on all” strategy. This significantly enhances the precision and cost-effectiveness of clinical decision-making.

## 4 Discussion

IgAV is predominantly an immune-mediated small-vessel disease, pathologically defined by IgA deposition. Up to 50% of patients may develop occult or overt gastrointestinal bleeding. The abdominal phenotype presents unique diagnostic challenges, as the temporal relationship between abdominal pain and skin purpura is often unpredictable, and symptoms are highly variable (3, 4). Patients with abdominal pain and bloody stools may be misdiagnosed as having hemorrhagic necrotizing enteritis, while those exhibiting isolated right lower quadrant pain may erroneously be diagnosed with acute appendicitis (4). Early gastrointestinal involvement in IgAV can disrupt cell membranes and impair tissue perfusion. This initial ischemia is thought to stem from dysregulated immune responses and an imbalanced cytokine milieu (4, 6).

In this study, we used high-throughput Olink proteomics to compare plasma inflammatory profiles between abdominal IgAV patients and healthy controls. We observed significant upregulation of 22 cytokines, including IL-8, OSM, TGF-α, TNFSF-14, and HGF. This finding aligns with prior reports linking aberrant cytokine secretion to disease recurrence, gastrointestinal injury, and renal damage in IgAV. However, earlier work has largely been fragmentary; systematic biomarker discovery in abdominal IgAV remains underexplored. Below, we discuss the biological roles of these candidates and their potential clinical implications.

IL-8 is a chemokine that recruits and activates neutrophils, thereby directly influencing vessel wall inflammation. Its pathogenic role has been established in several autoimmune small-vessel vasculitides (15). OSM, a member of the IL-6 cytokine family, is implicated in multiple inflammatory conditions. It promotes endothelial activation and inflammatory cell infiltration via JAK–STAT and MAPK pathways (16). This process can amplify cytokine secretion NF-κB activation, perpetuating inflammation (1). TNFSF-14 (LIGHT), a member of the tumor necrosis factor superfamily, exerts dual effects on T-cell activation and apoptosis. Dysregulation of TNFSF-14 has been linked to rheumatoid arthritis, gastric cancer, and inflammatory bowel disease. This cytokine signals through three receptors—HVEM, LTβR, and DcR3, with distinct functional outcomes. Binding to HVEM delivers co-stimulatory signals to T cells, promoting proliferation and IFN-γ production (17). The upregulation of TNFSF-14 in IgAV may reflect immune dysregulation, particularly in pathways governing cell growth and differentiation. Furthermore, it has been implicated in angiogenesis and immune cell activation (4, 6). TGF-α and HGF primarily contribute to tissue repair, cell proliferation, and migration, suggesting concurrent injury and repair processes in IgAV (18).

Unlike earlier studies that focused solely on classical inflammatory markers, our data reveal a broader, coordinated upregulation of multiple cytokines. This observation suggests that the inflammatory response in IgAV is not linear but rather involves a complex, multi−pathway network (19).

Compared to other small-vessel inflammatory conditions, IgAV exhibits a distinctive cytokine signature. Notably, IL-8 and OSM expression levels differ significantly from those observed in IgA nephropathy, wherein IL-8 is considerably lower (20, 21). This discrepancy indicates a unique immune phenotype in IgAV, where specific cytokines are elevated disproportionately and may directly influence clinical presentation and prognosis. The interaction between TNFSF-14 and IL-8 could further amplify inflammation, leading to more severe symptoms. These observations underscore the need for cytokine-targeted strategies in the management of IgAV.

Validation through ELISA strengthens our findings. By confirming elevated levels of IL-8, OSM, and TNFSF−14 at the protein level in an independent, larger cohort, we mitigate concerns regarding false positives from batch effects or experimental design (22). ELISA is both sensitive and specific for inflammatory cytokines and is readily adaptable for clinical use (23). Moreover, while many previous biomarker studies in IgAV relied on urine or tissue samples, our work uses plasma, which is easier to obtain and more directly reflects systemic inflammation (24). This wet-lab validation interlinks high-throughput screening with clinical translation.

The ROC analysis of the combined IL-8/OSM/TNFSF-14 panel showed individual AUCs of 0.755, 0.817, and 0.861, respectively, and a combined AUC of 0.922, indicating excellent diagnostic accuracy. This combination significantly outperformed any single marker, a trend similarly observed in other pediatric systemic vasculitis studies (25). Notably, the three biomarkers identified within this study, IL-8, TNFSF-14, and OSM, do not function independently but act synergistically in the pathogenesis of the abdominal phenotype of IgAV. This relationship delineates a sequential and interactive inflammatory pathway that interlinks innate immune activation, adaptive immune dysregulation, and tissue destruction. IL-8 acts as the primary neutrophil chemoattractant responsible for the recruitment and infiltration of neutrophils into the intestinal microvasculature, triggering endothelial damage and initiating the early inflammatory cascade characteristic of IgAV vasculitis. TNFSF-14 further modulates T-cell activation, differentiation, and tissue infiltration, enhancing adaptive immune responses and perpetuating intestinal vascular inflammation (26). Sustained inflammatory stimulation subsequently promotes the release of OSM, mediating extracellular matrix degradation, mucosal barrier disruption, and tissue remodeling, ultimately leading to abdominal pain, intestinal injury, and the typical clinical features of the abdominal phenotype (27). This coordinated pathogenic cascade strongly supports our conclusion that the three-marker panel provides a more comprehensive and integrated understanding of IgAV pathogenesis than individual biomarkers alone, thereby offering a more complete picture of disease mechanisms underlying abdominal involvement (28, 29).

DCA findings further support the clinical value of this panel. Previous IgAV diagnostic models have largely relied on clinical parameters and routine lab tests; few have incorporated high−dimensional molecular data. Thus, our model not only expands the methodological toolkit for IgAV diagnosis but also lays the foundation for more precise, mechanism−based diagnostic approaches.

Regarding clinical applicability, majority of clinically acceptable risk thresholds (10%–85%), using this nomogram model for risk stratification can preempt 2 to 25 unnecessary interventions or adverse events per 100 patients treated, compared to an “intervene on all” approach. This significantly enhances the precision and cost-effectiveness of clinical decision-making.

Functional enrichment analysis revealed that the upregulated proteins cluster within cytokine-cytokine receptor interaction pathways and the NF-κB signaling cascade. NF-κB functions as a master regulator of inflammation; its activation drives the expression of multiple downstream cytokines, thereby exacerbating endothelial damage and immune cell infiltration. This cascade is central to IgAV pathology (30). The cytokine-receptor network bridges innate and adaptive immunity, regulates leukocyte adhesion and migration, and modulates vascular permeability, offering a mechanistic understanding of the clinical features of abdominal IgAV (13, 29). Compared to pathway analyses in other autoimmune vasculitides (e.g., SLE, Kawasaki disease), the inflammatory network in IgAV places greater emphasis on NF-κB and its effectors. This finding extends prior single-factor studies and provides a rationale for targeting NF-κB or its upstream regulators in future therapeutic development (31, 32).

Several limitations of this study warrant attention in future work. First, a small sample size may lead to type II errors and overfitting of the ROC curve. Although IL-8, OSM, and TNFSF-14 exhibited strong performance within our cohort, their sensitivity and specificity across larger, more diverse populations require validation. As this study was an exploratory prospective study, we plan to enhance the sample size and undertake validation in subsequent cohort studies. Second, the single-center, retrospective design limits the generalizability of the biomarker panel. Multicenter prospective studies are needed to validate these findings and assess diagnostic performance across broader populations and clinical settings. Third, the lack of a disease control group precludes a comprehensive evaluation of the panel’s specificity for abdominal-type IgAV. Lastly, based on the findings of this clinical study, our team will subsequently conduct related basic experiments, such as cell and animal modeling, to further conduct multi-dimensional scientific exploration pertaining to the onset and progression of IgAV. Future studies should incorporate control patients with clinically similar conditions such as acute surgical abdomen or inflammatory bowel disease to more accurately evaluate the specificity of this panel for abdominal-type IgAV.

## 5 Conclusion

This study identified IL-8, OSM, and TNFSF-14 as promising plasma biomarkers for IgAV. These findings open new avenues for improving early diagnosis and risk stratification in this challenging clinical population. Furthermore, these insights provide a molecular foundation for understanding IgAV pathogenesis and designing targeted therapeutic approaches. Moving forward, large-scale, multicenter studies are needed to validate the diagnostic utility of this biomarker panel and to explore its relationship with clinical outcomes and treatment responses.

## Data Availability

The original contributions presented in the study are included in the article/supplementary material. Further inquiries can be directed to the corresponding author.
